# Lung Ultrasound-Guided Fluid Management versus Standard Care in Surgical ICU Patients: A Randomised Controlled Trial

**DOI:** 10.3390/diagnostics11081444

**Published:** 2021-08-10

**Authors:** Daniel-Mihai Rusu, Ioana Grigoraș, Mihaela Blaj, Ianis Siriopol, Adi-Ionut Ciumanghel, Gigel Sandu, Mihai Onofriescu, Olguta Lungu, Adrian Constantin Covic

**Affiliations:** 1 Anaesthesia and Intensive Care Department, Grigore T. Popa University of Medicine and Pharmacy, 700115 Iasi, Romania; rusu.daniel.ro@gmail.com (D.-M.R.); mihaela.blaj@umfiasi.ro (M.B.); ianismarian-sn-siriopol@umfiasi.ro (I.S.); olguta.lungu@umfiasi.ro (O.L.); 2 Anaesthesia and Intensive Care Department, Regional Institute of Oncology, 700483 Iasi, Romania; 3 Anaesthesia and Intensive Care Department, Sf. Spiridon University Hospital, 700111 Iasi, Romania; adi.ionut80@yahoo.com (A.-I.C.); gigelsandu@ymail.com (G.S.); 4 Nephrology Department, Grigore T. Popa University of Medicine and Pharmacy, 700115 Iasi, Romania; onomihai@yahoo.com (M.O.); adrian.covic@umfiasi.ro (A.C.C.); 5 Nephrology Department, Dr. C.I. Parhon University Hospital, 700503 Iasi, Romania

**Keywords:** lung ultrasound, B-line score, extravascular lung water, fluid management, intensive care, randomised controlled trial

## Abstract

The value of lung ultrasound (LU) in assessing extravascular lung water (EVLW) was demonstrated by comparing LU with gold-standard methods for EVLW assessment. However, few studies have analysed the value of B-Line score (BLS) in guiding fluid management during critical illness. The purpose of this trial was to evaluate if a BLS-guided fluid management strategy could improve fluid balance and short-term mortality in surgical intensive care unit (ICU) patients. We conducted a randomised, controlled trial within the ICUs of two university hospitals. Critically ill patients were randomised upon ICU admission in a 1:1 ratio to BLS-guided fluid management (active group) or standard care (control group). In the active group, BLS was monitored daily until ICU discharge or day 28 (whichever came first). On the basis of BLS, different targets for daily fluid balance were set with the aim of avoiding or correcting moderate/severe EVLW increase. The primary outcome was 28-day mortality. Over 24 months, 166 ICU patients were enrolled in the trial and included in the final analysis. Trial results showed that daily BLS monitoring did not lead to a different cumulative fluid balance in surgical ICU patients as compared to standard care. Consecutively, no difference in 28-day mortality between groups was found (10.5% vs. 15.6%, *p =* 0.34). However, at least 400 patients would have been necessary for conclusive results.

## 1. Introduction

Despite increasing awareness of the deleterious effects of fluid overload (FO) [1] and the advances made in guiding fluid therapy [2,3,4,5], avoiding FO in intensive care unit (ICU) patients remains challenging. Consequently, the number of patients with positive fluid balance (FB) and FO during ICU stay is still worryingly high [6]. FO leads to tissue and organ oedema [7] and has been associated with increased risk of postoperative complications [8], acute kidney injury (AKI) [9], prolonged mechanical ventilation [10], and prolonged ICU and hospital length of stay (LOS) [11,12]. Moreover, a meta-analysis summarizing current evidence related to FO’s impact on mortality in 31 observational studies reported an increased risk of mortality in the general ICU population with FO or positive cumulative FB (CFB) [1]. FO is multifactorial [13], but the challenge of finding the right moment to start fluid de-escalation is a major contributor; for instance, clinical examination, FB, chest X-ray, and patients’ oxygen requirements are often used by clinicians to trigger fluid de-escalation over more reliable (but more invasive) volume assessment methods [14]. In this context, the possible value of monitoring extravascular lung water (EVLW) with lung ultrasound (LU) in order to individualise fluid management and improve outcome has recently come into question [15].

EVLW increase is an early marker of pulmonary oedema [15]; thus, its assessment may be used to limit fluid administration or trigger fluid de-escalation, as pulmonary oedema may be further worsened in the context of a positive FB. Transpulmonary thermodilution is the method currently used for EVLW measurement [16]. This method requires central venous and arterial cannulation, time, expertise, and resources [17,18], and is usually reserved for the most complex ICU cases. Computed tomography (CT) and nuclear magnetic-performed resonance imaging (MRI) may also be used for EVLW assessment [19,20]. However, despite their great value in diagnosing several pulmonary and extrapulmonary conditions, CT and MRI scans of the chest are impractical for daily monitoring of EVLW as they are costly, time-consuming, and they expose patients to transportation hazards or high doses of radiation. LU can detect increases in EVLW [21,22] and its dynamic changes [23,24] noninvasively at the bedside, with minimal distress for the patient and using minimal resources [25]. B-lines are the ultrasonographic signs of EVLW increase [26]. The close correlation between the number of B-lines on LU and EVLW volume has already been demonstrated by comparing LU with gold-standard methods for EVLW evaluation [27,28,29]. Moreover, B-lines can be easily detected [30] using various ultrasound systems and probes [31] with good intra- and inter-evaluator reliability [32,33,34,35]. Nonetheless, LU is infrequently used to guide fluid therapy, as its added value in fluid management is still a matter of debate.

This study’s primary aim was to evaluate the impact of a B-Line score (BLS) fluid management strategy on ICU patients’ short-term mortality. Our central hypothesis was that the daily assessment of BLS, coupled with active fluid removal in cases of moderate or severe EVLW increase (as reflected by the BLS value), might improve CFB and decrease 28-day mortality as compared to standard care. The secondary hypotheses were that BLS-guided fluid management would decrease 90-day mortality, ICU and hospital LOS, AKI recovery time, and the duration of vasopressor therapy and mechanical ventilation.

## 2. Materials and Methods

### 2.1. Study Design and Settings

From November 2017 to November 2019, we conducted a randomised, controlled trial within two tertiary hospitals’ ICUs to determine whether BLS-guided fluid management could decrease 28-day mortality in critically ill patients, as compared to standard care. The study was approved by the Research Ethics Committees of the Grigore T. Popa University of Medicine and Pharmacy Iași (No 26261/14 November 2017) and was conducted under the principles of the Declaration of Helsinki. Written informed consent was obtained from all subjects/legal representatives.

The trial was retrospectively registered on ClinicalTrials.gov (https://clinicaltrials.gov/ct2/show/NCT03393065) accessed on 8 January 2018.

The study protocol has been published elsewhere [36].

### 2.2. Participants

During the trial, study investigators performed a daily screening of all ICU admissions to identify patients who fulfilled one of the following inclusion criteria: major surgery, major comorbid conditions in surgical patients, polytrauma with an Injury Severity Score (ISS) ≥ 15, an Acute Physiology and Chronic Health Evaluation II (APACHE II) score on admission ≥ 10 or a Sequential Organ Failure Assessment (SOFA) score on admission ≥ 6. Major surgery included: Esophagectomy, Total Gastrectomy, Total Colectomy, Duodeno-pancreatectomy, Major Hepatectomy, Multi-Organ Resection, Aorto-Bifemoral Bypass, Aortic Interposition Tube Graft. Major comorbid conditions included: Chronic Obstructive Pulmonary Disease (COPD) Global Initiative for Obstructive Lung Disease (GOLD) stage III or IV, Heart Failure New York Heart Association (NYHA) Class III or IV, Heart Valve Disease grade III or IV, Cirrhosis Child-Pugh score B or C, Chronic Kidney Disease (CKD) stage 1–4.

Patients fulfilling any of the following criteria were excluded: trial participation refusal, age < 18 years, pregnancy, known pulmonary conditions that can interfere with LU interpretation (pneumectomy, pulmonary fibrosis, pulmonary lymphangitis, persistent pleural effusion), CKD stage 5, or indication for emergency renal replacement therapy (RRT), previous prolonged resuscitation (≥10 min) for cardiorespiratory arrest.

### 2.3. Randomisation and Blinding

After enrolment, patients were randomly assigned to BLS-guided fluid management (active group) or standard care (control group) in a 1:1 ratio, using block randomisation. The randomised sequence was created using a computerised random-number generator and concealed at the coordinating centre. The allocation group was provided each time a new patient was enrolled using a 24-h phone service. Patients and healthcare providers were not blinded, but the outcome assessors were blinded to the patient’s group assignment. Data analysis was performed before the allocation sequence code was broken.

### 2.4. Lung Ultrasound Performance

**Figure 1:**
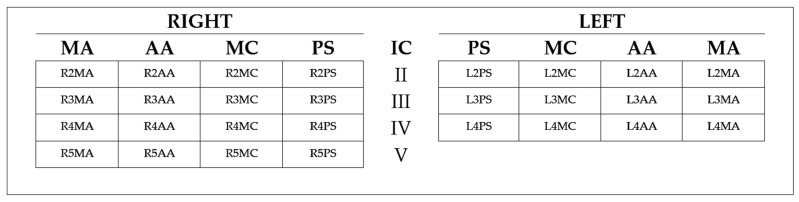
A map of chest site scans for BLS assessment. The figure shows the 28 chest sites scanned for the BLS calculation. The code of each site describes its space alignment: R: Right Chest; L: Left Chest; 1 to 5: the number of the intercostal space (IC); MA: Mid-Axillary Line; AA: Anterior-Axillary Line; MC: Mid-Clavicular Line; PS: Parasternal Line.

In the active group, LU was performed daily, from ICU admission to ICU discharge, or day 28 (whichever came first). In the control group, LU was only performed once on admission, and the treating physician remained blinded to LU data. All LU examinations were made by trained ICU physicians at the bedside, with the patient in the supine position; the focus of the image was set at the pleural line level and the depth of penetration set between 40 and 80 mm. The ultrasound equipment used was the GE LOGIQ V2^®^ ultrasound system and the GE 3Sc-RS Cardiac Sector Probe^®^ (General Electric Healthcare, Chicago, IL, USA). The BLS assessment protocol consisted of a complete scan of 28 chest sites, as described by Jambrik et al. [37]. The sum of all B-lines seen on LU defined the BLS. A map of chest sites scans is provided in Figure 1.

### 2.5. Fluid Management

In the active group, with every LU examination, patients were stratified into four classes: no EVLW increase (BLS = 0–4), mild increase (BLS = 5–14), moderate increase (BLS = 15–29), or severe EVLW increase (BLS ≥ 30), based on BLS severity grading system proposed by Frassi et al. [38]. In patients with no or mild EVLW increase (BLS = 0–14), a zero FB was targeted if no signs of shock were present. In patients with a moderate or severe increase in EVLW (BLS ≥ 15), a daily negative FB of –250 to –1000 mL was targeted until BLS dropped under 15. To reach daily targeted FB, furosemide-induced diuresis and RRT were used. Furosemide was administrated in a stepwise manner considering the previous furosemide dose and the FB achieved. If the targeted FB was achieved from the first day of diuretic administration, the furosemide dose was maintained. If FB was outside the targeted range, the furosemide dose was progressively reduced or increased until the goal was achieved. RRT was used in patients with moderate and severe EVLW increase (BLS ≥ 15) if the targeted FB could not be reached despite using the maximum furosemide dose of 800 mg/day. Outside trial interventions, overall ICU patients’ management was at the treating physicians’ discretion.

In the control group, fluid management was guided by the Enhanced Recovery after Surgery (ERAS) principles. Within the ERAS protocol, the aim was to maintain an adequate intravascular volume while minimising weight gain. Various parameters were used to attain this goal based on case-by-case clinical judgment: lung sounds, heart rate, blood pressure, temperature, urine output, FB, lactate, haemoglobin, haematocrit, serum urea, creatinine, sodium, potassium, chloride, and bicarbonate values. Additionally, central venous oxygen saturation, pulse pressure variation and stroke volume variation were used to assess fluid responsiveness in patients with shock.

**Figure 2:**
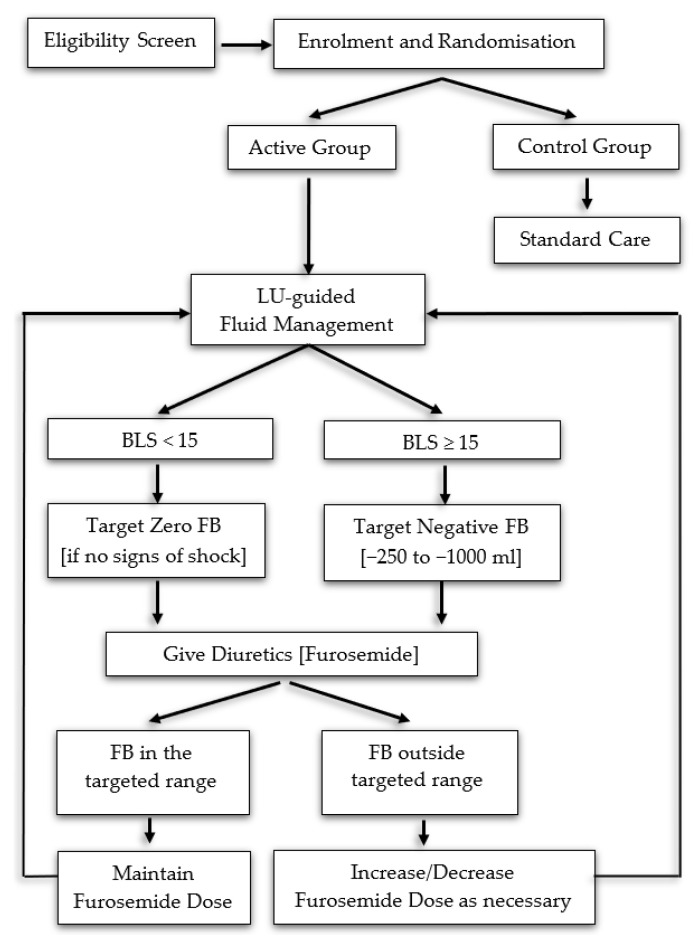
Trial algorithm.

The trial algorithm is presented in Figure 2. The recommended furosemide regimens are provided in Table 1.

### 2.6. Collected Variables

Data were collected from the ICU charts and hospital medical records. Survival was assessed via a phone call to the patient or the patient’s legal representative. On ICU admission, age, gender, body mass index (BMI), primary diagnosis, surgery type, infectious status, organ dysfunctions, comorbid conditions, severity scores, BLS, and main laboratory data were collected. During the ICU stay, data regarding fluid management, BLS (active group), organ dysfunctions and organ support therapies were collected. Outcome data were 28-day and 90-day mortality, ICU and hospital LOS, AKI, vasopressor therapy, and mechanical ventilation duration.

### 2.7. Study Sample Size

We estimated that, with a sample size of 199 patients in each group, the study would have 80% power to detect an absolute difference of 10% in the primary outcome, assuming a 28-day mortality rate of 20% in the control group, at a two-sided 5% level of significance. The choice of 10% expected difference in the primary outcome was based on mortality rates in patients with and without FO, observed in a large cohort study of ICU patients [11]. To account for potential withdrawals of consent, the recruitment target was set at 250 patients in each arm. For circumstantial reasons (the COVID-19 pandemic), the study was not able to reach the targeted sample size.

### 2.8. Statistical Analysis

Data were analysed using MedCalc Statistical Software version 19.1.7 (MedCalc Software Ltd., Ostend, Belgium, 2020). All analyses were conducted on an intention-to-treat basis. The intention-to-treat population was formed by all trial participants, except those who withdrew consent. No assumptions for missing data were made. Variables distribution was tested for normality using histograms and the Shapiro-Wilk test. Comparisons between continuous variables were performed using Student’s *t*-test (for normally distributed data) or Mann-Whitney U-test (for non-normally distributed data). Comparisons between categorical variables were performed using Chi-square (χ2) test or Fisher’s exact test, as appropriate. Hazard ratios (HRs) and risk ratios (RRs) with 95% confidence intervals (CIs) were used to evaluate the effect size of BLS-guided fluid management on the primary outcome and 90-day mortality. Cohen’s kappa and Cliff’s delta statistics were used for estimating the effect size (ES) of active vs. control group allocation on continuous secondary outcomes.

An exploratory analysis of the effect of active group allocation on the primary outcome was performed across non-prespecified subgroups of patients.

Continuous variables are presented as means and standard deviations (sd) if normally distributed or as medians and 25–75% interquartile ranges (IQRs) if non-normally distributed. Categoric variables are presented as number (*n*) and percentage (%). Data are presented by group allocation. For all analyses, a *p*-value < 0.05 was considered statistically significant.

## 3. Results

### 3.1. Study Patients

**Figure 3:**
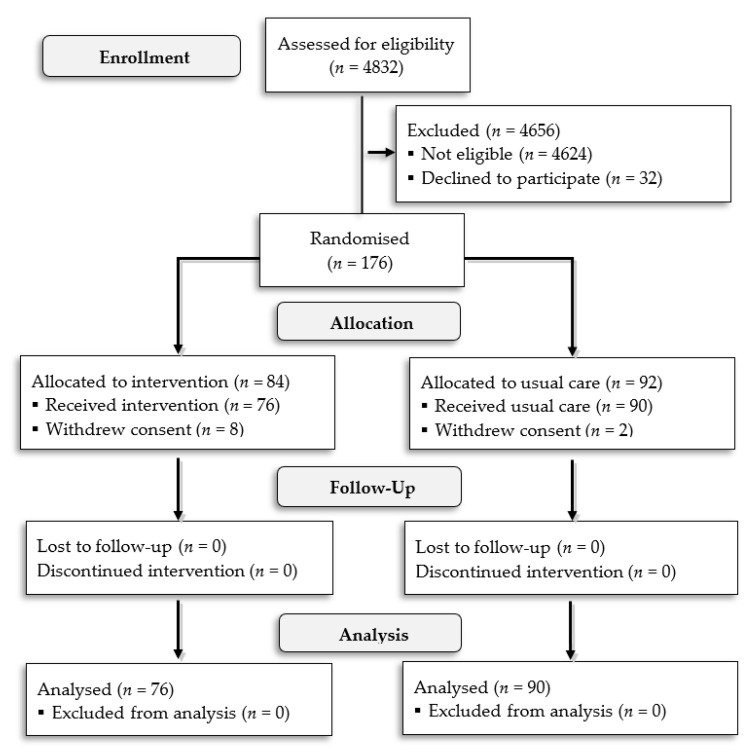
CONSORT diagram showing patient flow through the trial.

Over the study period, 208 patients were eligible, based on the inclusion criteria. Informed consent was obtained from 176 patients who were further randomised in a 1:1 ratio to intervention or standard care. A total of 10 patients withdrew consent after randomisation. Hence, 166 patients were included in the final analysis. Patients flow through the trial is presented in a Consolidated Standards of Reporting Trials (CONSORT) diagram in Figure 3. 

### 3.2. Baseline Data

The median age of the study population was 64 (IQR 59–70) years. The male-female ratio was 2:1. Mean BMI was 25.6 (sd 4.2) kg/m^2^. The majority of patients (162 patients, 97.6%) were admitted to the ICU following surgery: 30 (18.1%) patients after emergency surgery and 132 (79.5%) patients after elective surgery. The primary diagnosis was cancer in 112 (67.5%) patients. Forty-one (24.7%) patients had sepsis or septic shock. Ninety-five (57.2%) patients had organ dysfunction. The leading comorbidities were cardiovascular diseases (117 patients, 70.5%), diabetes mellitus (48 patients, 28.9%) and CKD (42 patients, 25.3%). Anaemia was present in 145 (87.3%) patients. Hyperchloremia and hypokalaemia were the main imbalances found in serum electrolytes. Moderate/severe EVLW volume increase, as reflected by the BLS value, was observed on ICU admission in 32 (19.3%) patients. The median APACHE II score on admission was 8.5 (IQR 7–12), and the median SOFA score was 4 (IQR 2–6). The trial arms were well balanced, with no significant differences in baseline characteristics between groups. See Table 2 and Table 3.

### 3.3. Lung Ultrasound Data and Fluid Balance

**Figure 4:**
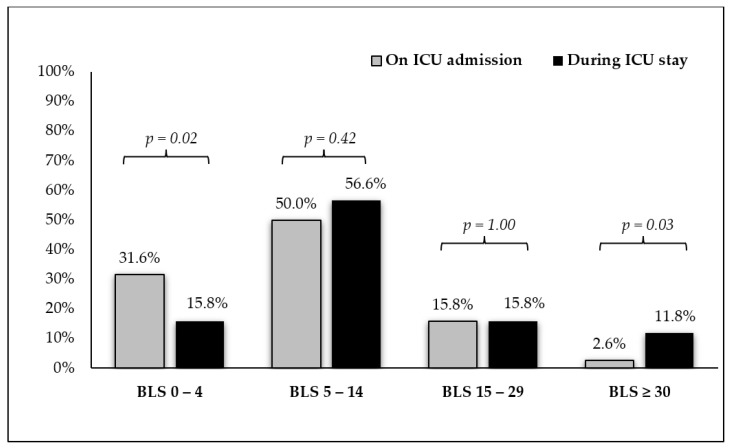
B-Line score dynamics in the active group. The figure shows the percentages of patients with no (BLS = 0–4), mild (BLS = 5–14), moderate (BLS = 15–29), and severe (BLS ≥ 30) EVLW increase on ICU admission and during ICU stay. Abbreviations: BLS: B-Line score; EVLW: Extravascular Lung Water; ICU: Intensive Care Unit.

Four hundred forty LU exams were performed in the active group, with an average of six exams/patient. Cohen’s kappa for the inter-rater agreement was 0.91 (95% CI 0.84 to 0.98). From all of the LU exams, 97 (22%) did not reveal signs of EVLW increase (BLS < 5), 231 (52.5%) revealed a mild EVLW increase (BLS = 5–14), 79 (11.9%) revealed a moderate increase (BLS = 15–29), and 33 (7.5%) revealed a severe EVLW increase (BLS ≥ 30). Following admission, the number of patients without LU signs of EVLW increase (BLS < 5) dropped from 24 (31.6%) to 12 (15.8%) (*p* = 0.02), while the number of those with severe EVLW increase (BLS ≥ 30) rose from 2 (2.6%) to 9 (11.8%) (*p* = 0.03); see Figure 4. Overall, of the 76 patients in the active group, 67 (88.2%) had LU signs of EVLW increase (BLS ≥ 5) at least once during their ICU stay, of which 23 (30.3%) demonstrated a moderate/severe EVLW increase (BLS ≥ 15).

Fluid de-escalation measures, defined as furosemide prescription to obtain a zero or negative FB, were taken early in both groups, and by ICU discharge, more than 90% of patients received furosemide at least once, with no major differences between trial arms (Table 4). The cumulative furosemide dose at ICU discharge was similar in both groups (120 vs. 110 mg, *p* = 0.74). Two patients (2.6%) from the active group and four from the control group (4.4%) required RRT to rebalance their volume status (*p* = 0.53).

The percentages of patients with zero or negative CFB at 24 h, 48 h, 72 h, and at ICU discharge were not significantly different between the active and control group. Similarly, there were no significant differences regarding the median CFB at 24 h, 48 h, 72 h, and at ICU discharge between the active and control group (Table 4).

### 3.4. Outcome Data

During the trial, there was no cross over between the groups and all the patients completed follow-up. The primary outcome analysis showed no significant difference in 28-day mortality in the active vs. control group (10.5% vs. 15.6%, *p* = 0.34, RR 0.68, 95% CI 0.30 to 1.53). Mean survival time by day 28 was similar in the two trial arms (26 vs. 25 days, HR 0.66, 95% CI 0.28 to 1.52, reference control group, *p* = 0.33). Secondary outcomes analyses revealed no significant differences between the active and control group in 90-day mortality (11.8% vs. 17.8%, *p* = 0.29, RR 0.67, 95% CI 0.31 to 1.42), ICU LOS (4 vs. 4 days, *p* = 0.78), hospital LOS (12 vs. 10 days, *p* = 0.17), AKI recovery time (6 vs. 5 days, *p* = 0.22), or vasopressor therapy duration (3 vs. 3 days, *p* = 0.97). We noticed that the hours on mechanical ventilation were significantly lower in the active vs. the control group (22 vs. 44 h, ES 0.67, *p* = 0.02), but ventilator-free days were not significantly different (26 vs. 20 days, *p* = 0.32). Mean survival time by day 90 was 81 days in the active group and 76 days in the control group (HR 0.64, 95% CI 0.29 to 1.42, reference control group, *p* = 0.28). Outcome data are presented in Table 5.

In the explorative analyses, we found that the BLS-guided fluid management effect on the primary outcome was significantly different across subgroups of patients with emergency surgery and sepsis/septic shock. The results showed a decreased mortality in emergency surgery patients and patients with sepsis/septic shock that received BLS-guided fluid management in the postoperative period as compared with the standard care group (Table 6).

## 4. Discussion

To our knowledge, this is the first study to analyse the potential outcome effects of a fluid management strategy based on BLS assessment in a population of surgical critically ill patients. The concept of using B-lines dynamics to guide fluid therapy is not new. It was developed in 2012 by Lichtenstein, who proposed fluid administration limited by lung ultrasonography (the FALLS protocol) in patients with acute circulatory failure [39]. However, this concept has never been tested in a controlled clinical trial. Our study showed no significant difference in the short-term mortality of patients receiving BLS-guided fluid management and those receiving standard care. The 90-day mortality, ICU and hospital LOS, duration of vasopressor therapy and AKI recovery time were similar in the two trial arms. We noticed a significantly lower mechanical ventilation duration in the active vs. control group, but the overall ventilator-free days were not significantly different between groups.

Several other studies that compared restrictive or active fluid de-escalation strategies with standard care reported similar results. In the Fluid and Catheter Treatment Trial, 1000 patients with acute lung injury were randomised to a conservative or a liberal fluid strategy [40]. The trial reported a decreased mechanical ventilation duration in the conservative group, but no significant difference in the 60-day mortality [40]. In a single-centre randomised trial, Richard et al. analysed the effects of fluid administration based on preload dependence indices in 60 septic shock patients [41]. In this study, no significant differences were noted regarding the 28-day mortality, ICU LOS, and vasopressor therapy duration in the preload dependence group as compared to standard care group [41]. Chen and Kollef randomised 82 septic shock patients under vasoactive therapy following the initial fluid resuscitation phase to fluid management guided by daily assessments of fluid responsiveness or standard care [42]. Their study results showed no significant differences regarding in-hospital mortality and vasopressor therapy duration between groups [42]. Moreover, Chen and Kollef did not report a significant difference in mechanical ventilation duration between groups [42]. Hjortrup et al. analysed a conservative fluid strategy effect on outcome vs. standard care in 151 septic shock patients [43]. They found no differences in the 90-day mortality between groups, but a higher risk for worsening AKI in the control group [43]. Recently, in a randomised pilot study, Corl et al. compared a restrictive fluid resuscitation strategy with standard care in 109 patients with severe sepsis and septic shock and found similar 30-day mortality rates, ICU and hospital LOS, and duration of vasopressors use [44]. The duration of ventilatory support was shorter in the restrictive fluid group, but the number of patients requiring new mechanical ventilation was only 32 [44].

In our study, a possible explanation for the lack of significant difference in CFB and short-term mortality between groups is that we examined BLS-guided fluid management within the context of ERAS pathways. Thus, increased efforts to attain a zero or negative FB were taken in both groups. These efforts are reflected in the increased number of patients that received diuretics early in their ICU stay, in both groups. Moreover, no significant difference was found between cumulative furosemide doses used in the active and control group. A recent survey of ICU physicians’ practices also pointed towards the increased use of several preventive and treatment strategies to attain a zero FB in standard care [45]. A few years ago, fluids were often prescribed in patients who develop hypotension, and ICU physicians were less willing to give diuretics to patients requiring vasopressors. At present, they favour the use of vasopressors for isolated hypotension treatment while continuing fluid removal in patients with signs of FO [45]. It also seems that in the presence of FO, ICU physicians target a negative 24 h FB of −500 to −1500 mL [45].

Another possible explanation is that we used the same diuretic protocol in all patients, while the varying situations that led to EVLW increase would have required different diuretic regimens to attain the same therapeutic response.

Finally, we enrolled a heterogeneous population of critically ill surgical patients in which B-lines may not have reflected only pulmonary congestion. While some studies point towards an accurate evaluation of EVLW with the BLS [46], others suggest that the accuracy of EVLW assessment with BLS depends on the studied population [47]. For instance, Seibel et al., in a large heterogeneous population of critically ill patients, found that the correlation coefficient between BLS on LU and EVLW assessed by transpulmonary thermodilution was highly variable, with the highest sensitivity and specificity of BLS to predict EVLW increase being in the subgroup of patients with a PaO_2_/FiO_2_ ratio <200 mmHg [47]. Furthermore, in a recent study, Buda et al. showed that subpleural small consolidations might be responsible for at least some of the B-lines seen on LU [48]. Interestingly, by increasing ultrasound frequency from 2 MHz to 6 MHz, Buda et al. showed that most B-lines caused by lung surface abnormalities convert to Z-lines [48]. Mutually, most patients (97%) in whom B-lines were converted to Z-lines by increasing ultrasound frequency had pleural line abnormalities [48]. These recent findings indicate that the lung surface should also be checked for abnormalities in order to differentiate between the different possible aetiologies of B-lines [48]. In this context, our study results may reflect the futility of adding BLS data in fluid management decision-making without using additional parameters to increase BLS specificity. Future studies should establish whether the BLS alone yields any value or needs to be correlated with B-lines distribution patterns, cardiac ultrasound, PaO_2_/FiO_2_ ratio, or other parameters to increase its specificity.

The lower duration of mechanical ventilation observed in the active group is the single positive outcome in our study. However, in our trial, the number of patients on mechanical ventilation was too small to support any strong conclusions. It is possible that the clinicians felt more confident to extubate patients earlier simply by having a daily LU evaluation, but this may also be a spurious finding. If LU can indeed help decrease the duration of mechanical ventilation, it deserves further investigation in the future.

In the explorative analyses, we found a lower mortality in emergency surgery patients and patients with sepsis/septic shock that received LU-guided fluid management in the postoperative period. These hypotheses should be verified in future trials.

### Study Strengths and Limitations

Our results must be interpreted in light of certain strengths and limitations. The primary limitation is the lack of power, as a result of failing to achieve the desired sample size. Nevertheless, many other trials investigating different fluid management strategies have similar or smaller cohorts. A second limitation concerns the criteria used for participant selection. We targeted the recruitment of patients prone to positive FB, or even FO, following major surgery. We also enrolled surgical patients with major comorbidities, in which fluid retention may worsen their condition due to impaired respiratory, cardiovascular, kidney, or hepatic function. These inclusion criteria led to a heterogeneous study population that may have altered the results. Another limitation is that ICU physicians in the study were not blinded to patient group assignment, thus we cannot exclude the Hawthorne effect. Being aware that their patient’s outcome will be assessed, ICU physicians may have increased their efforts to improve FB in the control group by limiting the amount of prescribed fluids or by prescribing higher doses of diuretics.

A lack of clear guidelines for education in clinical LU [49] and a lack of consensus on the various methodological aspects and scoring systems used to assess EVLW [31] are common limitations of any fluid management protocol design based on LU, including ours. Several studies report that B-lines can be accurately identified by examiners with different levels of expertise [30,33,50], using different types of ultrasound machines and probes [31]. However, counting B-lines may be challenging [51]. In our trial, trained ICU physicians performed all LU exams, and the inter-rater agreement was high. Moreover, all patients were examined in a supine position using the same cardiac sector probe. Current evidence shows that patient positioning may influence LU findings, at least in patients with heart failure [52]. Regarding probe selection for B-lines detection, no advice is provided in the 2012 consensus guideline [53]. Nevertheless, according to various reports, the cardiac and convex probes are slightly more accurate in B-lines detection than other types of ultrasound transducers [54,55,56]. To quantify the degree of pulmonary congestion, we used a comprehensive approach, in which 28 predefined points were scanned for B-lines. This approach, first used by Jambrik et al. [37], seems to have a better diagnostic accuracy for EVLW assessment than others [57]. However, we did not analyse the relationship between B-lines and lung surface abnormalities, which might have caused at least some of the B-lines seen on LU [48].

In our study, different targets for daily FB were set using a BLS cut-off value of 15. This cut-off value was based on the severity grading system used by Frassi et al. to show the correlation between different BLS classes and survival in patients with dyspnoea, chest pain, or both [38]. In another study, Zoccali et al. reported an increased risk of mortality in end-stage CKD patients with a BLS ≥ 15 [58]. More recently, Yin et al. found that a high BLS on ICU admission was associated with an increased 28-day mortality in critically ill patients [59]. On the basis of these findings, we assumed that maintaining a BLS < 15 would improve the outcome. However, it is not clear what “the safe range” is for BLS in a surgical ICU population and how BLS should be maintained in that range to improve outcome.

In the active group, we used a protocol for fluid removal to attain the targeted FB. The protocol was based on diuretic therapy and ultrafiltration. In patients with moderate and severe EVLW increase (BLS ≥ 15), the aim was to obtain a predefined −250 to −1000 mL/24 h negative FB until BLS dropped under 15. However, this strategy may not be appropriate for all patients, as some evidence indicates an average decrease of only 2.7 B-lines per 500 mL fluid removed [23]. Furthermore, we did not use LU to assess fluid responsiveness and titrate fluid input, but only to count B-lines and establish daily FB targets. A combined approach might have been more efficient in affecting FB and outcome.

In the explorative analyses of the primary outcome, the subgroups were not prespecified. Therefore, the risk of spurious findings should not be ignored.

Finally, we believe that important LU data may be lost when summarised in a single value such as the BLS. This needs to be highlighted as bedside LU is rapidly becoming a highly valued method for EVLW assessment. Learning to identify B-lines is easy, and previous studies showed a quick learning curve [60]. Moreover, other studies showed promising results regarding automatic B-line detection using image-processing algorithms [61]. However, the majority of the studies that found LU-guided fluid management to be associated with lower CFB as compared to standard care used LU in combination with cardiac ultrasound or inferior vena cava ultrasonography [62,63,64]. Thus, the utility or futility of BLS assessment may depend on additional ultrasound data, which should be considered when designing LU-based fluid management strategies.

## 5. Conclusions

Within its limitations, this trial suggests that daily BLS monitoring, with the aim of avoiding and correcting moderate/severe EVLW increase, does not improve CFB in surgical ICU patients compared to standard care. Moreover, it does not improve 28-day or 90-day survival, ICU or hospital LOS, AKI recovery time, or duration of vasopressors use. Daily BLS assessment might help decrease mechanical ventilation duration, but this result needs to be verified in a larger trial. Future studies should also establish whether BLS alone yields any value in a heterogeneous population of critically ill surgical patients or needs to be correlated with B-lines distribution patterns, cardiac ultrasound, PaO_2_/FiO_2_ ratio, or other parameters to increase its specificity in EVLW assessment.

## Figures and Tables

**Table 1:** The recommended Furosemide regimens to attain targeted fluid balance.

Previous Furosemide Dose (mg/day)		Recommended Furosemide Dose (mg/day)
1	≤80 mg	40 mg iv bolus + 5 mg/h
2	81–160 mg	80 mg iv bolus + 10 mg/h
3	161–240 mg	80 mg iv bolus + 20 mg/h
4	>240 mg	80 mg iv bolus + 30 mg/h

**Table 2:** Baseline characteristics of patients.

Variable	Active Group (*n* = 76)	Control Group (*n* = 90)	*p*
**Age** (years)	63.5 (58–71)	64.5 (60–70)	0.94
**Male Gender**	51 (67.1)	63 (70.0)	0.69
**BMI** (kg/m^2^)	25.8 (3.9)	25.4 (4.5)	0.58
**Surgery**			
Emergency	14 (18.4)	16 (17.8)	0.91
Elective	62 (81.6)	70 (77.8)	0.55
**Primary diagnosis**			
Cancer	52 (68.4)	60 (66.7)	0.81
Nononcologic Disease	24 (31.6)	30 (33.3)	0.81
**Comorbid Conditions**	65 (85.5)	77 (85.6)	0.99
COPD/Asthma	12 (15.8)	8 (8.9)	0.17
Cardiovascular Diseases	54 (71.0)	63 (70.0)	0.88
Hepatitis/Cirrhosis	10 (13.2)	14 (15.6)	0.66
CKD	19 (25.0)	23 (25.6)	0.81
Previous Stroke	4 (5.3)	3 (3.3)	0.54
Diabetes Mellitus	21 (27.6)	27 (30.0)	0.74
Autoimmune Diseases	1 (1.3)	4 (4.4)	0.24
Major comorbidities	22 (28.9)	32 (35.6)	0.37
**Sepsis/Septic shock**	20 (26.3)	21 (23.3)	0.66
**Organ dysfunction**	43 (56.6)	52 (57.8)	0.88
**Anaemia**	64 (84.2)	81 (90.0)	0.26
**Electrolytes’ imbalances**			
Hyperkalaemia	1 (1.3)	0 (0.0)	0.40
Hypokalaemia	10 (13.2)	14 (15.6)	0.62
Hypernatremia	6 (7.9)	7 (7.8)	0.98
Hyponatremia	2 (2.6)	4 (4.4)	0.53
Hyperchloremia	16 (21.0)	18 (20.0)	0.87
Hypochloraemia	1 (1.3)	1 (1.1)	0.90
**EVLW on LU**			
normal (BLS 0–4)	24 (31.6)	29 (32.2)	0.93
mild increase (BLS 5–14)	38 (44.7)	43 (47.8)	0.78
moderate increase (BLS 15–29)	12 (15.8)	10 (11.1)	0.38
severe increase (BLS ≥ 30)	2 (2.6)	8 (8.9)	0.09
**Severity Scores**			
Charlson Comorbidity Index	6.4 (2.7)	6.0 (2.2)	0.32
APACHE II	8 (6–12)	9 (7–14)	0.08
SOFA	4 (2–6)	4 (1–7)	0.61

Data are given as number (%), mean (standard deviation) or median (quartile 25–75%). Abbreviations: APACHE II: Acute Physiology and Chronic Health Evaluation II score; BLS: B-Line score; BMI: Body Mass Index; CKD: Chronic Kidney Disease; COPD: Chronic Obstructive Pulmonary Disease; EVLW: Extravascular Lung Water; SOFA: Sequential Organ Failure Assessment score.

**Table 3:** Surgical procedures performed on the studied patients.

Surgery Type	Active Group (*n* = 76)	Control Group (*n* = 90)	*p*
Esophagectomy	2 (2.6)	1 (1.1)	0.46
Total Gastrectomy	18 (23.7)	15 (16.7)	0.26
Duodeno-pancreatectomy	11 (14.5)	21 (23.3)	0.15
Hepatectomy	5 (6.6)	4 (4.4)	0.55
Total Colectomy	1 (1.3)	0 (0.0)	0.28
Multi-organ Resection	10 (13.2)	11 (12.2)	0.86
Aorto-bifemoral Bypass	11 (14.5)	13 (14.4)	0.99
Aortic interposition tube graft	2 (2.6)	2 (2.2)	0.86
Damage control surgery	14 (18.4)	16 (17.8)	0.91
Other type	2 (2.6)	3 (3.3)	0.79

Data are given as number (%).

**Table 4:** Fluid de-escalation measures and CFB across groups.

Variable	Active Group	Control Group	*p*
**Fluid De-escalation**, pts (%)			
day 1	57/76 (75.0)	71/90 (78.9)	0.55
day 2	58/74 (78.4)	61/84 (72.6)	0.40
day 3	44/56 (78.6)	53/67 (79.1)	0.94
overall	70/76 (92.1)	82/90 (91.1)	0.82
**Cumulative Furosemide dose**, mg	120 (60–270)	110 (65–220)	0.74
**RRT**, pts (%)	2 (2.6)	4 (4.4)	0.53
**Zero or Negative CFB**, pts (%)			
at 24 h	21/76 (27.6)	21/90 (23.3)	0.53
at 48 h	18/74 (24.3)	20/84 (23.8)	0.94
at 72 h	14/56 (25.0)	23/67 (34.3)	0.26
at ICU discharge	21/76 (27.6)	29/90 (32.2)	0.52
**Median CFB**, mL			
at 24 h	630 (−139–1430)	680 (115–1366)	0.52
at 48 h	1320 (1–2406)	1379 (47–2157)	0.86
at 72 h	1580 (−56–3079)	1150 (−515–2593)	0.27
at ICU discharge	1027 (−374–2690)	1027 (−430–2875)	0.97

Data are given as the number of cases/total number of patients (%) or as median (quartile 25–75%). Abbreviations: CFB: Cumulative Fluid Balance; Pts: Patients; RRT: Renal Replacement Therapy.

**Table 5:** Primary and secondary outcomes.

	Group Allocation		RR/ES (95% CI)	*p*
	Active (*n* = 76)	Control (*n* = 90)		
**28-day mortality**	8 (10.5)	14 (15.6)	0.68 (0.30 to 1.53)	0.34
90-day mortality	9 (11.8)	16 (17.8)	0.67 (0.31 to 1.42)	0.29
ICU LOS, days	4 (2–6)	4 (2–6)	0.04	0.78
ICU-free days	24 (20–26)	24 (21–25)		0.62
Hospital LOS, days	12 (9–18)	10 (7–16)	0.23	0.17
Hospital-free days	76 (69–81)	77 (66–81)		0.84
Patients with AKI	11 (14.5)	23 (25.6)		0.08
AKI recovery time, days	6 (4–11)	5 (3–6)	0.43	0.22
AKI-free days	18 (3–23)	22 (0–24)		0.98
Patients on Vasopressors	40 (52.6)	48 (53.3)		0.93
Vasopressors use, days	3 (2–5)	3 (1–5)	0.01	0.97
Vasopressors-free days	25 (22–26)	25 (0–27)		0.57
Patients on MV	21 (27.6)	30 (33.3)		0.43
MV duration, hours	22 (6–48)	44 (22–107)	0.67	0.02
Ventilator-free days	26 (0–27)	20 (0–27)		0.32

Data are given as number (%) and RR (95% CI) or median (quartile 25–75%) and ES. Abbreviations: AKI: Acute Kidney Injury; CI: Confidence Interval; ES: Effect Size; LOS: Length-Of-Stay; MV: Mechanical Ventilation; RR: Risk Ratio.

**Table 6:** Primary outcome across non-prespecified subgroups.

	28-Day Mortality		RR (95% CI)	*p*
	Active	Control		
**Surgery**				
emergency	1/14 (7.1)	10/16 (62.5)	0.11 (0.02 to 0.78)	<0.01
elective	7/62 (11.3)	3/70 (4.3)	2.63 (0.71 to 9.75)	0.13
**Sepsis/septic shock**				
yes	1/20 (5.0)	8/21 (38.1)	0.13 (0.02 to 0.96)	0.01
no	7/56 (12.5)	6/69 (8.7)	1.44 (0.51 to 4.03)	0.49
**AKI or CKD**				
yes	3/25 (12.0)	12/37 (32.4)	0.37 (0.12 to 1.18)	0.06
no	5/51 (9.8)	2/53 (3.8)	2.60 (0.53 to 12.79)	0.22
**AHF or CHF**				
yes	7/46 (15.2)	14/55 (25.4)	0.60 (0.26 to 1.35)	0.21
no	1/30 (3.3)	0/35 (0.0)	3.48 (0.15 to 82.48)	0.28

Data are given as the number of patients with negative outcome/total number of exposed patients (%) and RR (95% CI). Abbreviations: AHF: Acute Heart Failure; AKI: Acute Kidney Injury; CHF: Chronic Heart Failure; CI: Confidence Interval; CKD: Chronic Kidney Disease; RR: Risk Ratio.

## Data Availability

The datasets used and/or analysed during the current study are available from the corresponding author on reasonable request.
